# Correction of collimator-dependent differences in the heart-to-mediastinum ratio in ^123^I-metaiodobenzylguanidine cardiac sympathetic imaging: Determination of conversion equations using point-source imaging

**DOI:** 10.1007/s12350-016-0546-8

**Published:** 2016-06-01

**Authors:** Yusuke Inoue, Yutaka Abe, Kei Kikuchi, Keiji Matsunaga, Ray Masuda, Kazutoshi Nishiyama

**Affiliations:** 1 Department of Diagnostic Radiology, Kitasato University School of Medicine, 1-15-1 Kitasato Minami-ku, Sagamihara, Kanagawa 252-0374 Japan; 2 Department of Radiology, Kitasato University Hospital, Sagamihara, Japan; 3 Department of Neurology, Kitasato University School of Medicine, Sagamihara, Japan

**Keywords:** ^123^I-metaiodobenzylguanidine (MIBG), collimator, heart-to-mediastinum (H/M) ratio, septal penetration

## Abstract

**Background:**

Septal penetration causes collimator-dependent differences in the heart-to-mediastinum (H/M) ratio in ^123^I-metaiodobenzylguanidine (MIBG) cardiac imaging. We investigated generally applicable methods to correct such differences.

**Methods and Results:**

Four hours after ^123^I-MIBG injection, 40 patients underwent anterior chest imaging successively with medium-energy (ME) and various non-ME collimators. The H/M ratios obtained with the non-ME collimators before and after ^123^I-dual-window penetration correction were compared with the ME-derived standard values to determine patient-based conversion equations for empiric and combined corrections, respectively. A ^123^I point source was imaged with various collimators, and the central ratio, the ratio of count in a small central region of interest to count in a large one, was calculated. The method of predicting the conversion equations from the central ratios was determined. Correction using the patient-based conversion equations removed systematic underestimation of the H/M ratios obtained with the non-ME collimators, and combined correction depressed residual random errors to some degree. Point-source-based equations yielded results comparable to the patient-based equations.

**Conclusions:**

Empiric and combined corrections effectively reduce collimator-dependent differences in the H/M ratio. The conversion equations can be predicted from simple point-source imaging, which would allow to apply these corrections to data obtained with various collimators.

**Electronic supplementary material:**

The online version of this article (doi:10.1007/s12350-016-0546-8) contains supplementary material, which is available to authorized users.

## Introduction

Cardiac sympathetic imaging with ^123^I-metaiodobenzylguanidine (^123^I-MIBG) is used for the assessment of disease severity and prognosis in heart failure^1^ as well as for the evaluation of neurodegenerative disorders such as Parkinson’s disease and dementia with Lewy bodies.^2^ The kinetics of ^123^I-MIBG in the left ventricle reflect sympathetic innervation, and patients with impaired cardiac sympathetic function exhibit decreased accumulation and accelerated washout. For quantitative assessment, the heart-to-mediastinum (H/M) ratio, the ratio of count density in the left ventricle to that in the upper mediastinum, is used extensively.

Collimator choice affects the estimation of the H/M ratio greatly, which has been reviewed elsewhere.^3^ Low-energy (LE) collimators are designed primarily for imaging ^99m^Tc sources, and are often applied to ^123^I imaging. Although an LE collimator is appropriate for 159-keV primary photons of ^123^I, this radionuclide also emits high-energy photons of more than 400 keV that easily penetrate the thin septa of an LE collimator. Septal penetration of high-energy photons degrades image quality and quantitative accuracy in ^123^I imaging. Medium-energy (ME) collimators have thicker septa than LE collimators and effectively prevent septal penetration. The use of an ME collimator improves quantitative accuracy in ^123^I imaging and provides better estimates of H/M ratios compared with an LE collimator.^4^-^6^ However, LE collimators are often applied to cardiac ^123^I-MIBG imaging because of their wide availability.^6^

Septal penetration causes systematic underestimation of the H/M ratios due to predominant overestimation of the mediastinum count^3^ and disturbs comparison of values obtained using different collimators. To facilitate interfacility comparison and intrafacility comparison before and after changing the imaging protocol, methods to correct collimator-dependent differences have been investigated. The triple-energy-window method to remove the effects of scattering and septal penetration failed to attain reliable correction in previous phantom studies.^4^,^5^ In other studies,^7^,^8^ patients were successively imaged with the ME and non-ME collimators, and regression equations between the H/M ratios obtained with different collimators were determined. Using the regression equation, the non-ME-derived H/M ratio was converted to an equivalent value to be obtained with the ME collimator. This correction method, termed empiric correction because of the empiric determination of the conversion equation, effectively removes systematic underestimation caused by septal penetration. However, imaging with a non-ME collimator makes estimation of the H/M ratio susceptible to the variability of surrounding radioactivity, and the degree of the underestimation depends on the intensity and distribution of activity in the liver and lung.^4^,^9^ Even after removal of systematic underestimation by empiric correction, random errors remain.^7^,^8^ The ^123^I-dual-window (IDW) correction is a penetration correction method in which the influence of septal penetration of high-energy photons is predicted based on counts in a high-energy subwindow.^10^ Although the IDW correction alone did not yield satisfactory results,^7^,^8^ a combined method of empiric correction and IDW correction achieved better correction than pure empiric correction: removal of systematic underestimation and concomitant reduction of random errors.^8^

A given vendor offers collimators of various specifications, and collimators of the same name provided by different vendors differ in actual specifications. Although the conversion equations should depend on detailed collimator specifications, the equation for combined correction was determined only for one collimator provided by one vendor in the previous study.^8^ It is troublesome to determine the equation for each collimator based on successive patient imaging. Convenient phantom-based determination of conversion coefficients has been proposed;^11^,^12^ however, apparent underestimation remained after the correction.^12^ Additionally, the method is a simple conversion like the empiric correction described above, and does not reduce random errors.

In this study, we compared the H/M ratios obtained with ME and various non-ME collimators in the same patients, and determined conversion equations for empiric correction and combined correction. Furthermore, using the results, we developed a novel method of predicting the conversion equations from point-source images to correct the H/M ratios obtained with collimators that were not examined in this study. Our aim was to establish generally applicable methods for correcting collimator-dependent differences of the H/M ratios.

## Materials and Methods

### Instruments

Two dual-headed gamma camera systems were used in this study: e.cam (Siemens, Erlangen, Germany) and BrightView X with XCT (Philips Medical Systems, Cleveland, OH). The low-energy high-resolution (LEHR), low-energy all-purpose (LEAP), special low-energy high-resolution (SLEHR), low-medium-energy (LME), and ME collimators were used for the Siemens camera, and the cardiac high-resolution (CHR) collimator was used for the Philips camera. The specifications of the collimators are presented in Table 1. The shape of the hole was hexagonal in all collimators. The pixel sizes were 2.398 and 1.199 mm using 256 × 256 and 512 × 512 matrices, respectively, for the Siemens camera; they were 2.332 and 1.166 mm using 256 × 256 and 512 × 512 matrices, respectively, for the Philips camera. Analyses of image data, including those obtained using the Philips camera, were performed on an e.soft workstation (Siemens).

**Table 1:** Specifications of the collimators

Vendor	Collimator	Hole length (mm)	Hole diameter (mm)	Septal thickness (mm)
Siemens	LEHR	24.05	1.11	0.16
Siemens	LEAP	24.05	1.45	0.20
Siemens	SLEHR	40.00	1.90	0.25
Siemens	LME	37.00	2.50	0.60
Siemens	ME	40.64	2.94	1.14
Philips	CHR	48.00	2.03	0.152

### Point-Source Imaging

A point source of ^123^I was imaged with each collimator to evaluate the degree of septal penetration. Approximately 30 MBq ^123^I-MIBG in 0.5-mL solution was put in a 2.5-mL plastic syringe, which was then placed on the imaging table of the gamma camera system, 10 cm below the center of the detector head. The radioactivity in the plastic syringe was measured using a dose calibrator (IGC-7; Hitachi Aloka Medical, Tokyo, Japan) and corrected for the effect of LE photons.^13^ The photopeak energy window was a 20% window centered at 159 keV (range, 143.1-174.9 keV), and the high-energy subwindow for IDW correction was a 50% window centered at 235 keV (range, 176.3-293.8 keV). The matrix was 512 × 512, the zoom factor was 1, and the acquisition time was 5 min. Background counts in the absence of radioactive sources were measured with each collimator using the same imaging parameters.

Small and large square regions of interest (ROIs) were set such that the centers of the ROIs corresponded to the center of the point source. The side length was 55 mm for the small ROI (46 and 47 pixels for the Siemens and Philips cameras, respectively) and 380 mm for the large ROI (317 and 326 pixels for the Siemens and Philips cameras, respectively). After background subtraction, the central ratio, defined as the total count in the small ROI to that in the large ROI, was calculated before and after penetration correction by the IDW method. The IDW-corrected count (C_IDW_) was calculated using the following equation:

1 $$ C_{\text{IDW}} = C_{\text{p}} - \frac{{C_{\text{s}} }}{{W_{\text{s}} }} \times W_{\text{p}}, $$

where *C*_p_ and *C*_s_ are the total counts in the photopeak window and subwindow, respectively, and *W*_p_ and *W*_s_ are the widths of the photopeak window and subwindow, respectively. For the calculation of the IDW-corrected central ratio, both counts in the small and large ROIs were corrected by the IDW method.

### Patient Imaging

Forty patients (24 men and 16 women; mean age ± SD, 71.4 ± 9.6 years) who underwent cardiac ^123^I-MIBG imaging for the evaluation of neurodegenerative disorders were enrolled in this study. The study protocol was approved by the institutional review board. All patients provided written informed consent prior to participating in the study.

The patients underwent anterior planar imaging of the chest at the early (15 min) and late (4 h) phases after the injection of ^123^I-MIBG at a dose of 147.3 ± 2.9 MBq. The injection dose was measured in the glass syringe. The matrix was 256 × 256 in patient imaging, and other imaging parameters were the same as the point-source imaging described above.

Late images were acquired successively with three different collimators in each patient. The subjects were divided into two groups: groups A (n = 20) and B (n = 20). In group A, the LEAP and SLEHR collimators were used in addition to the ME collimator. Ten patients were imaged with the LEAP, ME, and SLEHR collimators, in that order, and the remaining 10 patients were imaged in the reverse order. In group B, the LME and CHR collimators were used in addition to the ME collimator. Ten patients were imaged with the LME, ME, and CHR collimators, in that order, and the remaining 10 patients in the reverse order. In both groups, the first, second, and third imagings were started approximately 3 h 50 min, 4 h, and 4 h 10 min after injection, respectively. Patients lay still on the imaging table during the collimator change for successive imagings with the Siemens camera, and moved to the next room for imaging with different cameras. Patients were not imaged with the LEHR collimator, and the conversion equations reported previously^8^ were used for analysis.

### Calculation of H/M Ratios

In processing of the Siemens camera images, ROIs for the heart and upper mediastinum were placed on the photopeak image obtained with the ME collimator and were copied onto other images. An irregular-shaped heart ROI was drawn manually over the left ventricle. The mediastinum ROI was set by a semiautomated method. The operator placed a rectangular preliminary ROI of 25 × 30 pixels to include the low-count area of the upper mediastinum. Then, the position of the final rectangular ROI of 12 × 20 pixels was determined automatically within the preliminary ROI to minimize the mean count in the final ROI. In processing of the Philips camera images, heart ROIs were drawn manually, visually referring to the ROIs drawn on the Siemens camera images of the same patient. The sizes of the preliminary and final ROIs were changed to 26 × 31 and 12 × 21 pixels, respectively, to compensate for the differences in pixel size between the two cameras. The H/M ratio, the ratio of the mean count density in the heart ROI to the mean count density in the mediastinum ROI, was calculated before and after IDW correction. The uncorrected H/M ratio measured with the ME collimator was regarded as a standard value.

### Determination of Conversion Equations

Based on the results of patient imaging, we determined the equations to convert the H/M ratios measured with the non-ME collimators to ME-based equivalent values (patient-based conversion equations). The standard values, i.e., uncorrected H/M ratios obtained with the ME collimator, were plotted against the ratios obtained with another collimator before and after IDW correction, and linear regression analysis was performed to determine the conversion equations for empiric correction and combined correction (combination of IDW correction and empiric correction), respectively.

Next, we investigated the methods to predict the conversion equations for empiric and combined corrections based on point-source data (point-source-based conversion equations). Regarding empiric correction, we substituted 4 for the ME-based equivalent value in the patient-based conversion equation for empiric correction, to calculate the uncorrected H/M ratio equivalent to the ME-based value of 4 (uncorrected 4-equivalent) for each collimator. If the equation to convert a non-ME-based H/M ratio (*x*) to an ME-based value (*y*) was *y* = *ax* + *b* (*a* and *b* are constants), we substituted 4 for *y* and calculated the 4-equivalent as (4 − *b*)/*a*. For the LEHR collimator, the conversion equation reported previously were used.^8^ The uncorrected 4-equivalents with various collimators were plotted against the uncorrected central ratios, and monoexponential curve fitting was performed to determine the equation to predict the uncorrected 4-equivalents from the uncorrected central ratio. Similarly, uncorrected H/M ratio equivalent to the ME-based value of 1 (uncorrected 1-equivalent) was calculated, and the equation to predict the uncorrected 1-equivalent from the uncorrected central ratio was determined. To predict the point-source-based conversion equation for empiric correction for a collimator, the uncorrected 4-equivalent and uncorrected 1-equivalent were calculated by substituting the uncorrected central ratio of the collimator in the monoexponential equations. Assuming the linear relationship between the ME-based and non-ME-based H/M ratios, the conversion equation was defined as follows:

2 $$ {\text{y}} = \frac{3}{{E_{4} - E_{1} }}x + \frac{{E_{4} - 4E_{1} }}{{E_{4} - E_{1} }}, $$

where *E*_4_ and *E*_1_ are the 4-equivalent and 1-equivalent, respectively.

In predicting the conversion equations for combined correction, the patient-based conversion equations for combined correction were used to calculate the IDW-corrected 4-equivalents and 1-equivalents. They were compared with the IDW-corrected central ratios, instead of the uncorrected central ratios.

The patient-based empiric correction, patient-based combined correction, point-source-based empiric correction, and point-source-based combined correction were applied to the patient imaging data obtained with the non-ME collimators. For empiric and combined corrections, the uncorrected and IDW-corrected H/M ratios measured with various collimators were substituted in the conversion equations, respectively. The non-ME-derived H/M ratios determined before and after correction were compared with the standard values by linear regression. The error was calculated as the non-ME-derived H/M ratio minus the standard value.

### Statistical Analysis

Values are expressed as mean ± SD. Linear regression analysis was performed using the least-squares method to assess the relationships between two variables. The absolute values of the errors were compared by paired *t* test between empiric and combined corrections. A *P* value less than 0.05 was deemed to indicate statistical significance.

## Results

Point-source imaging demonstrated counts in the peripheral regions of the field-of-view, distant from the point source, to various degrees depending on the collimators. The central ratio before IDW correction was the largest for the ME collimator, representing the least septal penetration, followed by the LME, SLEHR, LEAP, CHR, and LEHR collimators (Table 2). The IDW correction resulted in increases in the central ratios.

**Table 2:** Central ratios obtained by point-source imaging

Collimator	IDW (−)	IDW (+)
LEHR	0.476	0.667
LEAP	0.576	0.749
SLEHR	0.654	0.854
LME	0.843	0.939
ME	0.924	0.964
CHR	0.540	0.757

IDW (−) and IDW (+) indicate data before and after IDW correction, respectively

In patient imaging, underestimation of the H/M ratio was demonstrated for the non-ME collimators with no correction applied (Figures 1 and 2). The underestimation was more severe in patients with larger H/M ratios and small with the LME collimator. The mean H/M ratios were 2.18 ± 0.91, 1.71 ± 0.46, and 1.79 ± 0.54 with the ME, LEAP, and SLEHR collimators, respectively, in group A, and 2.07 ± 0.92, 1.96 ± 0.78, and 1.63 ± 0.44 with the ME, LME, and CHR collimators, respectively, in group B. The mean error was negative (Table 3). The underestimation was less severe for the LME collimator than for the other non-ME collimators.

**Figure 1:**
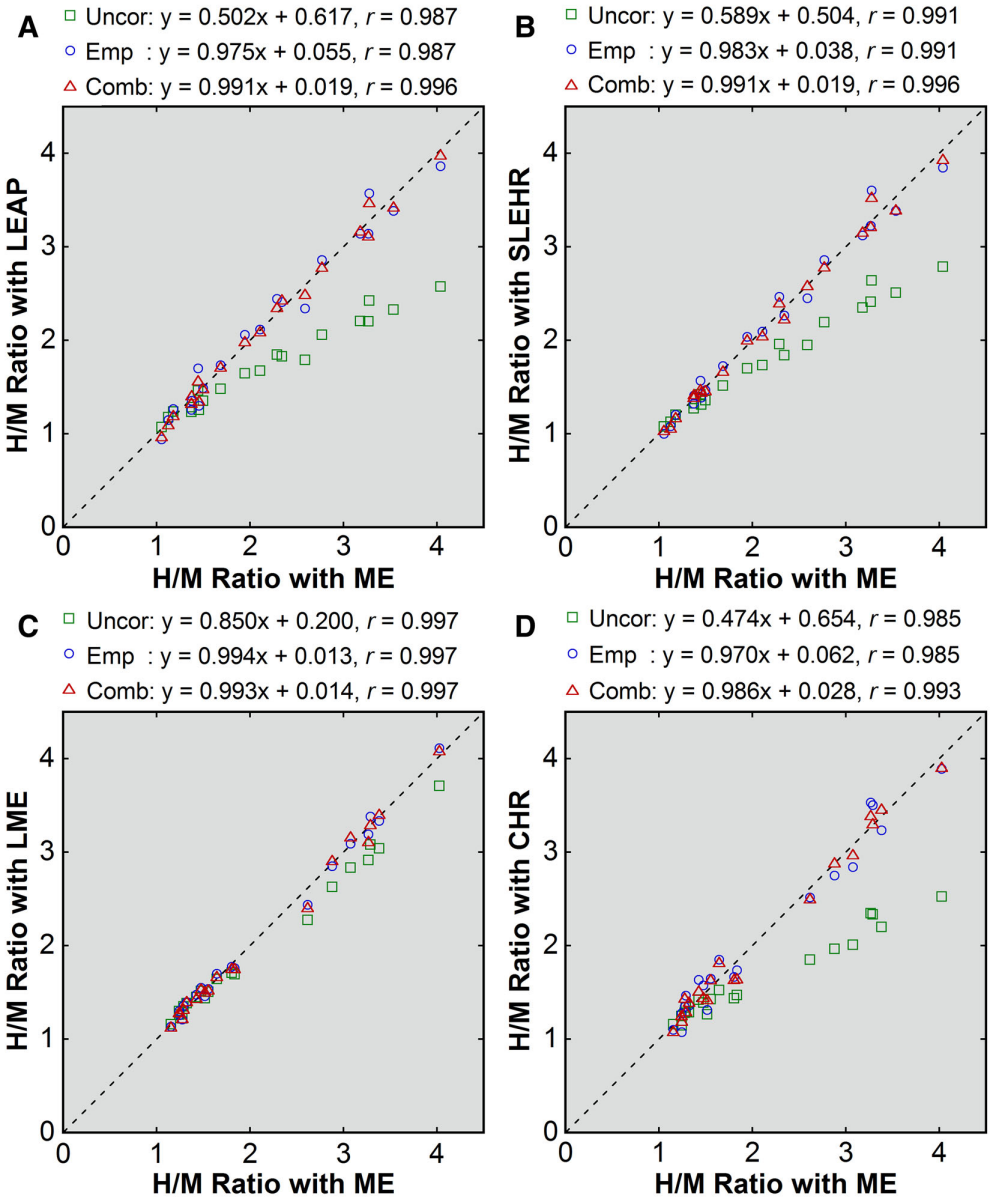
Patient-based correction of the H/M ratios obtained with non-ME (**A**, LEAP; **B**, SLEHR; **C**, LME; **D**, CHR) collimators. The values before correction (*green square*), after empiric correction (*blue circle*), and after combined correction (*red triangle*) were plotted against the uncorrected values obtained with the ME collimator. The *broken line* indicates the line of identity. Results of linear regression are shown

**Figure 2:**
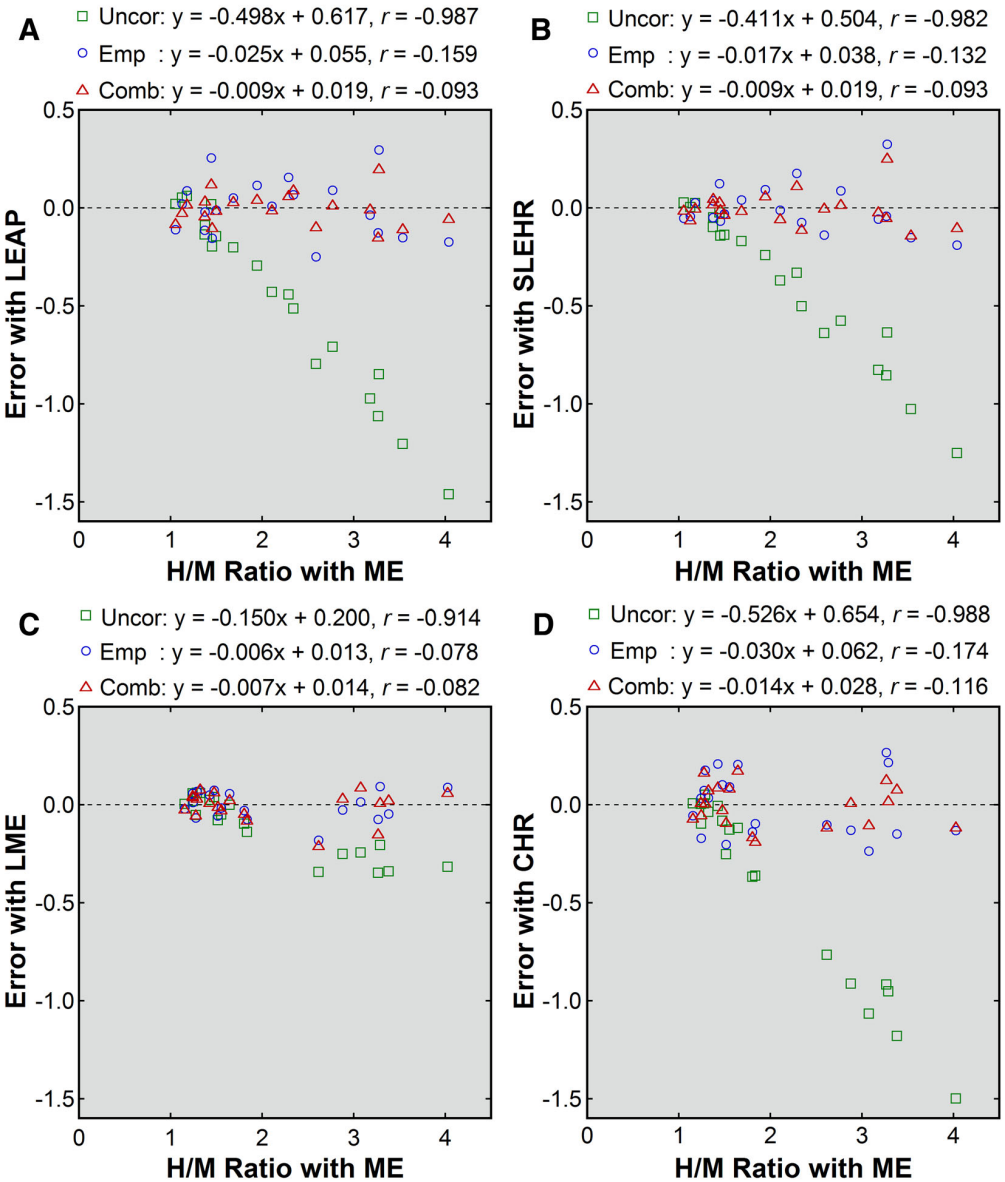
Errors before and after patient-based correction (**A**, LEAP; **B**, SLEHR; **C**, LME; **D**, CHR). Errors before correction (*green square*), after empiric correction (*blue circle*), and after combined correction (*red triangle*) were plotted against the uncorrected H/M ratios obtained with the ME collimator. Results of linear regression are shown

**Table 3:** Errors in the H/M ratio

Collimator	Error				
	No correction	Patient-based correction		Point-source-based correction	
		Empiric	Combined	Empiric	Combined
LEAP	−0.47 ± 0.46	0.00 ± 0.14	0.00 ± 0.08	0.01 ± 0.15	0.00 ± 0.09
SLEHR	−0.39 ± 0.38	0.00 ± 0.12	0.00 ± 0.09	−0.01 ± 0.12	0.00 ± 0.09
LME	−0.11 ± 0.15	0.00 ± 0.07	0.00 ± 0.08	0.00 ± 0.07	−0.01 ± 0.08
CHR	−0.43 ± 0.49	0.00 ± 0.16	0.00 ± 0.11	0.04 ± 0.16	0.01 ± 0.11

Values are mean ± SD

The patient-based conversion equations for empiric and combined corrections are presented in Table 4. These equations were used to calculate the uncorrected and IDW-corrected 4-equivalents and 1-equivalents for non-ME collimators. Regarding the ME collimator, the equation *y* = *x* was assumed for empiric correction, and the equation *y* = 0.920*x* + 0.097, determined from comparison between uncorrected and IDW-corrected ME-derived H/M ratios in group A, was used. The resulting uncorrected and IDW-corrected 4-equivalents increased with increasing uncorrected and IDW-corrected central ratios, respectively (Figure 3), and the monoexponential curve fitting was successful. The 1-equivalents were close to 1.

**Table 4:** Patient-based and point-source-based conversion equations for empiric and combined corrections

Collimator	Patient-based		Point-source-based	
	Empiric	Combined	Empiric	Combined
LEHR	*y* = 2.157*x* − 1.235	*y* = 1.428*x* − 0.453	*y* = 2.310*x* − 1.523	*y* = 1.438*x* − 0.490
LEAP	*y* = 1.943*x* − 1.143	*y* = 1.296*x* − 0.360	*y* = 1.876*x* − 1.019	*y* = 1.269*x* − 0.301
SLEHR	*y* = 1.668*x* − 0.803	*y* = 1.105*x* − 0.141	*y* = 1.615*x* − 0.719	*y* = 1.089*x* − 0.102
LME	*y* = 1.169*x* − 0.221	*y* = 0.976*x* + 0.020	*y* = 1.162*x* − 0.203	*y* = 0.967*x* + 0.031
CHR	*y* = 2.047*x* − 1.276	*y* = 1.225*x* − 0.244	*y* = 2.018*x* − 1.184	*y* = 1.253*x* − 0.284

The patient-based equations for the LEHR collimator are cited from Reference^8^

**Figure 3:**
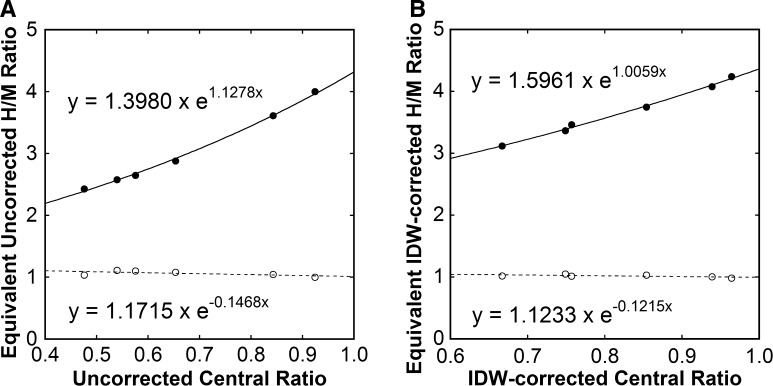
Central ratios and H/M ratios equivalent to the ME-based values of 4 (*closed circle*) and 1 (*open circle*) before (**A**) and after (**B**) IDW correction. Results of monoexponential curve fitting are shown

The uncorrected 4-equivalents and 1-equivalents were predicted from the equations in Figure 3A and the uncorrected central ratios, and the point-source-based conversion equations for empiric correction were determined (Table 4). The IDW-corrected 4-equivalents and 1-equivalents were predicted from the regression equations in Figure 3B and the IDW-corrected central ratios, and the point-source-based conversion equations for combined correction were determined (Table 4).

Corrections using the patient-based conversion equations effectively reduced collimator-dependent differences in the H/M ratios (Figures 1 and 2). Naturally, the systematic underestimation was removed, as indicated by the mean errors of zero (Table 3). After empiric correction, the SD of the error, representing residual random errors, was the largest for the CHR collimators, followed by the LEAP, SLEHR, and LME collimators. Combined correction mildly reduced the SD of the error for the LEAP, SLEHR, and CHR collimators when compared with empiric correction. For these three collimators, absolute values of the errors were significantly smaller after combined correction (LEAP, 0.06 ± 0.05; SLEHR, 0.06 ± 0.06; LME, 0.06 ± 0.05; CHR, 0.09 ± 0.06) than after empiric correction (LEAP, 0.12 ± 0.08, *P* < .001; SLEHR, 0.09 ± 0.08, *P* < .01; LME, 0.06 ± 0.04, *P* = .768; CHR, 0.14 ±  0.07, *P* < .05).

Point-source-based empiric and combined corrections also yielded successful results (Figures 4 and 5). The mean errors ranged from −0.01 to 0.04 for empiric correction and from −0.01 to 0.01 for combined correction, indicating almost complete removal of systematic underestimation (Table 3). The SD of the error was almost identical between patient-based and point-source-based correction.

**Figure 4:**
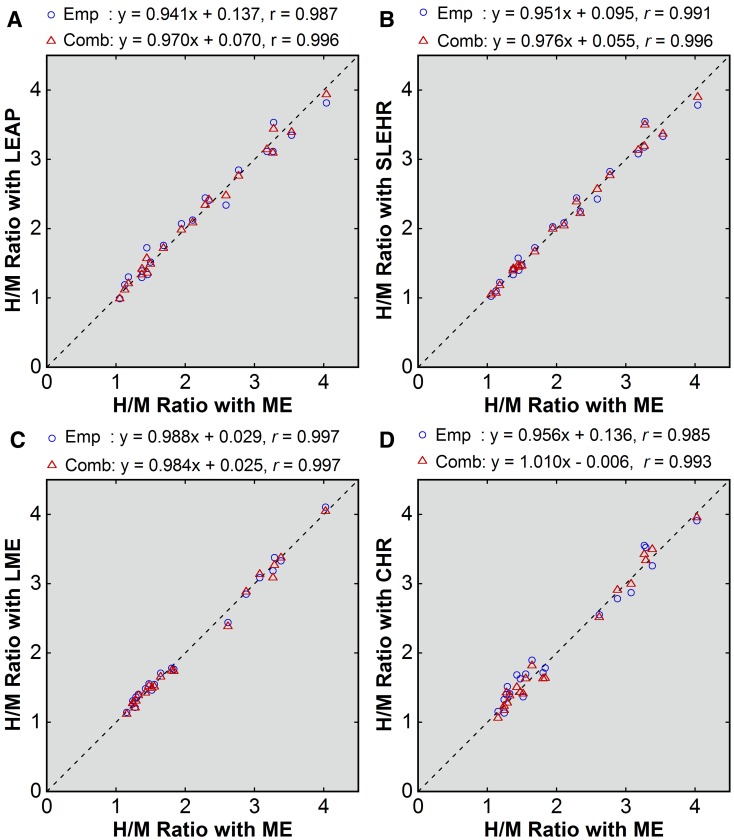
Point-source-based correction of the H/M ratios obtained with non-ME (**A**, LEAP; **B**, SLEHR; **C**, LME; **D**, CHR) collimators. The values after empiric correction (*blue circle*) and after combined correction (*red triangle*) were plotted against the uncorrected values obtained with the ME collimator. The *broken line* indicates the line of identity. Results of linear regression are shown

**Figure 5:**
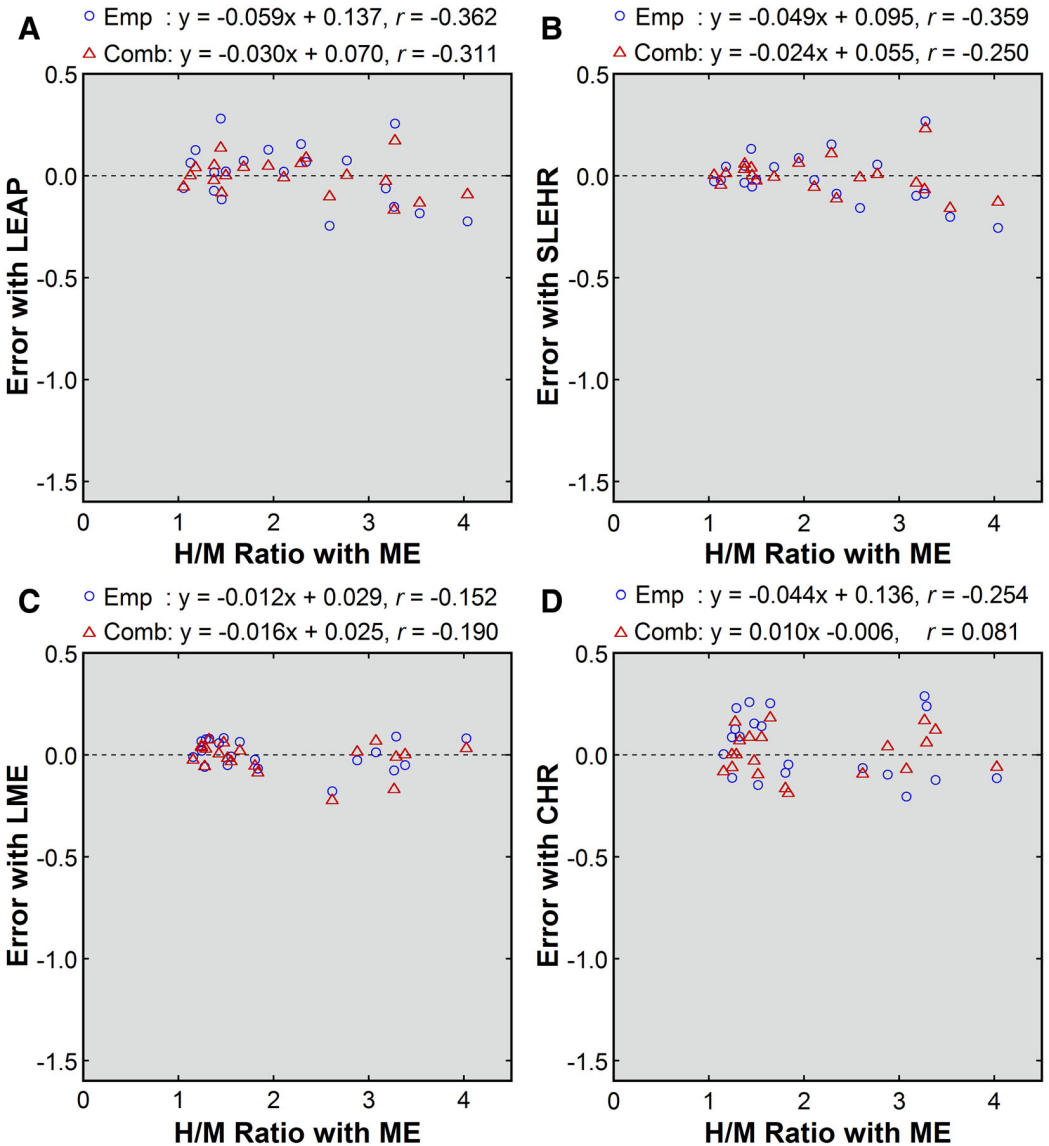
Errors after point-source-based correction (**A**, LEAP; **B**, SLEHR; **C**, LME; **D**, CHR) collimators. Errors after empiric correction (*blue circle*) and combined correction (*red triangle*) were plotted against the uncorrected H/M ratios obtained with the ME collimator. Results of linear regression are shown

## Discussion

In this study, we imaged the same patients successively with ME and various non-ME collimators, and calculated the H/M ratios. Using the results, we investigated generally applicable methods for estimating the H/M ratio to be obtained with the ME collimator from the images acquired with a non-ME collimator. Systematic underestimation of the H/M ratio, attributable to septal penetration, was confirmed for various non-ME collimators. More severe underestimation in patients with larger H/M ratios would disturb the assessment of the presence and severity of cardiac sympathetic function.^7^-^9^ Additionally, the intensity and distribution of ^123^I-MIBG activity in the lung and liver affect the degree of underestimation;^4^,^9^ and therefore, the non-ME-derived H/M ratios would reflect cardiac sympathetic function less faithfully than ME-derived values. The ME collimator is less susceptible to septal penetration and is recommended for estimation of the H/M ratio.^4^-^6^ However, when the ME collimator is not available, the conversion of non-ME-derived H/M ratios to ME-based values would be beneficial to facilitate comparisons of values obtained with different collimators.

In empiric correction, the H/M ratios obtained with a non-ME collimator are substituted in the conversion equation determined empirically to yield ME-based equivalent values. We determined the equations to convert non-ME-derived values to ME-based values by intrapatient comparisons of the ME-derived and non-ME-derived H/M ratios. Empiric correction removes systematic underestimation due to septal penetration; however, it does not reduce random errors.

The IDW method estimates the influence of septal penetration from images acquired in the high-energy subwindow. Although systematic errors in the H/M ratios remained after IDW correction alone in previous studies,^7^,^8^ the subwindow images reflect the patient-by-patient differences in the influence of penetration of high-energy photons; thus, the IDW correction has the potential to reduce not only systematic underestimation but also random errors. In combined correction, the IDW-corrected H/M ratios are converted to ME-based equivalent values using an empiric equation. The first step (IDW correction) decreases systematic underestimation and random errors, and then the second step (empiric correction) removes the remaining systematic underestimation. Superiority of this combination strategy to pure empiric correction was demonstrated for the LEHR collimator in a previous study^8^ and for the LEAP, SLEHR, and CHR collimators in the present study. The differences in residual errors between empiric and combined corrections were small for these three collimators, presumably because errors after empiric correction were small. However, *the differences in operator’s burden for data processing are also small*. We recommend the use of combined correction rather than pure empiric correction when counts in the same subwindow as that set in the present study are available. For the LME collimator, the combined correction did not provide additional benefit over the empiric correction, presumably because the underestimation with no correction was small and uncertainty in IDW correction canceled the potential minor benefit.

The conversion equations were established based on intrapatient comparison for five collimators as presented in Table 4. To predict the conversion equations for other collimators without additional intrapatient comparison, we investigated the relationship between the conversion equation and simple index reflecting the physical characteristics of the collimator. For convenience, point-source imaging was employed to assess the degree of septal penetration. The central ratio proposed here reflects counts associated with septal penetration, and its small value indicates that an image obtained with the collimator is highly susceptible to penetration. The H/M ratios equivalent to the ME-based value of 4, calculated for various collimators using the patient-based conversion equations, were strongly dependent on the central ratios. One of the collimators examined was provided by a vendor different from that for the other collimators; however, that collimator did not produce an outlier in the plots. Based on the relationship, the conversion equation was predicted from the central ratio. The application of correction using the point-source-based conversion equations achieved successful correction. The IDW-corrected central ratio appears to represent the influence of penetration on IDW-corrected patient images, and was used to predict the conversion equation for combined correction. Our point-source-based method of predicting conversion equations is easily feasible and permits correction of the H/M ratio with almost the same accuracy as patient-based correction. It appears to be valuable for enhancing the applicability of correction of collimator-dependent differences.

Point-source imaging to define the central ratios is quite simple; however, the imaging conditions should be the same as those used in the present study to apply the presented equations. Particularly, the importance of the source-collimator geometry appears to deserve special emphasis. When septal penetration is negligible, the distance affects spatial resolution, but its influence on counting sensitivity is limited. However, in imaging ^123^I with the non-ME collimator, the contribution of high-energy photons decreases at a larger distance, apparently reducing the counting sensitivity.^14^ Off-center positioning of the point source may also affect the estimation of the central ratios through varying counts in the peripheral region. It should be noted that the assessment of septal penetration is vulnerable to variations in the imaging conditions.

We summarize the strategy to correct collimator-dependent differences in the H/M ratio. If cardiac ^123^I-MIBG imaging is performed with one of the non-ME collimators described in Table 4, the patient-based conversion equation will be used. Combined correction is recommended for the Siemens LEHR, Siemens LEAP, Siemens SLEHR, and Philips CHR collimators if appropriate subwindow data are available, and empiric correction suffices for the Siemens LME collimator. For other collimators, the point-source-based conversion equations should be determined. First, point-source imaging is performed to measure the central ratio. If combined correction is planned, the IDW-corrected central ratio is substituted in the equations *y* = 1.5961 × e^1.0059*x*^ and *y* = 1.1233 × e^−0.1215*x*^ to calculate the IDW-corrected H/M ratios equivalent to the ME-based values of 4 (E_4_) and 1 (E_1_), respectively. If empiric correction is planned, the uncorrected central ratio is substituted in the equations *y* = 1.3980 × e^1.1278*x*^ and *y* = 1.1715 × e^−0.1468*x*^ to calculate the uncorrected H/M ratios equivalent to ME-based values of 4 and 1, respectively. The point-source-based conversion equation is determined as Eq. [2].

The use of an ME collimator is recommendable in cardiac ^123^I-MIBG imaging because it provides more accurate H/M ratios and also offers better SPECT images compared with an LEHR collimator.^15^ If an ME collimator is not available, a collimator with the least penetration in the facility may be used for imaging, followed by conversion to an ME-based equivalent value. The ME-based values cannot be compared directly with the cutoff values used in current clinical practice because the cutoff values appear to be determined from LE-based H/M ratios. Actually, the H/M ratios are supposed to have been obtained with various collimators provided by various vendors, resulting in various degrees of underestimation. To determine a new ME-based cutoff value, additional clinical trials are not needed. H/M ratios obtained in the previous studies can be easily converted to ME-based values by empiric correction using conversion equations determined for the respective collimators. Residual random errors after empiric correction would not cause substantial problems in determining a new cut-off value from group analysis.

## New Knowledge Gained

The conversion equations for empiric correction and combined correction determined based on patient data are presented to convert the H/M ratios obtained with various non-ME collimators to ME-based equivalent values. Combined correction, a combination of empiric correction and IDW correction, generally provides better accuracy than pure empiric correction. The conversion equations can be predicted from simple point-source imaging without patient imaging.

## Conclusions

In this study, we compared the H/M ratios obtained with various non-ME collimators with those obtained with the ME collimator, and determined conversion equations to correct collimator-dependent differences in the estimates of the H/M ratios. Underestimation of the H/M ratio, attributable to septal penetration, was confirmed for the non-ME collimators. Empiric correction using the patient-based conversion equations removed systematic underestimation, and the combined method of the empiric and IDW corrections depressed residual random errors, achieving more accurate correction to some degree. The conversion equations for empiric and combined corrections can be predicted from simple point-source imaging, which enhances the applicability of the correction methods. Although the use of the ME collimator is preferable, the correction of collimator-dependent differences presented here is expected to improve the usefulness of cardiac ^123^I-MIBG imaging.

## Electronic supplementary material

Below is the link to the electronic supplementary material.

Supplementary material 1 (PPTX 605 kb)

## Abbreviations

CHR: Cardiac high-resolution
IDW: ^123^I-dual-window
^123^I-MIBG: ^123^I-metaiodobenzylguanidine
H/M: Heart-to-mediastinum
LE: Low-energy
LEAP: Low-energy all-purpose
LEHR: Low-energy high-resolution
LME: Low-medium-energy
ME: Medium-energy
ROI: Region of interest
SLEHR: Special low-energy high-resolution
