# Supplementary material for: Correction of collimator-dependent differences in the heart-to-mediastinum ratio in 123I-metaiodobenzylguanidine cardiac sympathetic imaging: Determination of conversion equations using point-source imaging
Source: J Nucl Cardiol. 2016 Jun 1;24(5):1725–36. doi: 10.1007/s12350-016-0546-8 (PMC5629249; doi:10.1007/s12350-016-0546-8)
Supplement: Supplementary file 1 — Supplementary material 1 (PPTX 605 kb) [file 12350_2016_546_MOESM1_ESM.pptx]

## Slide 1
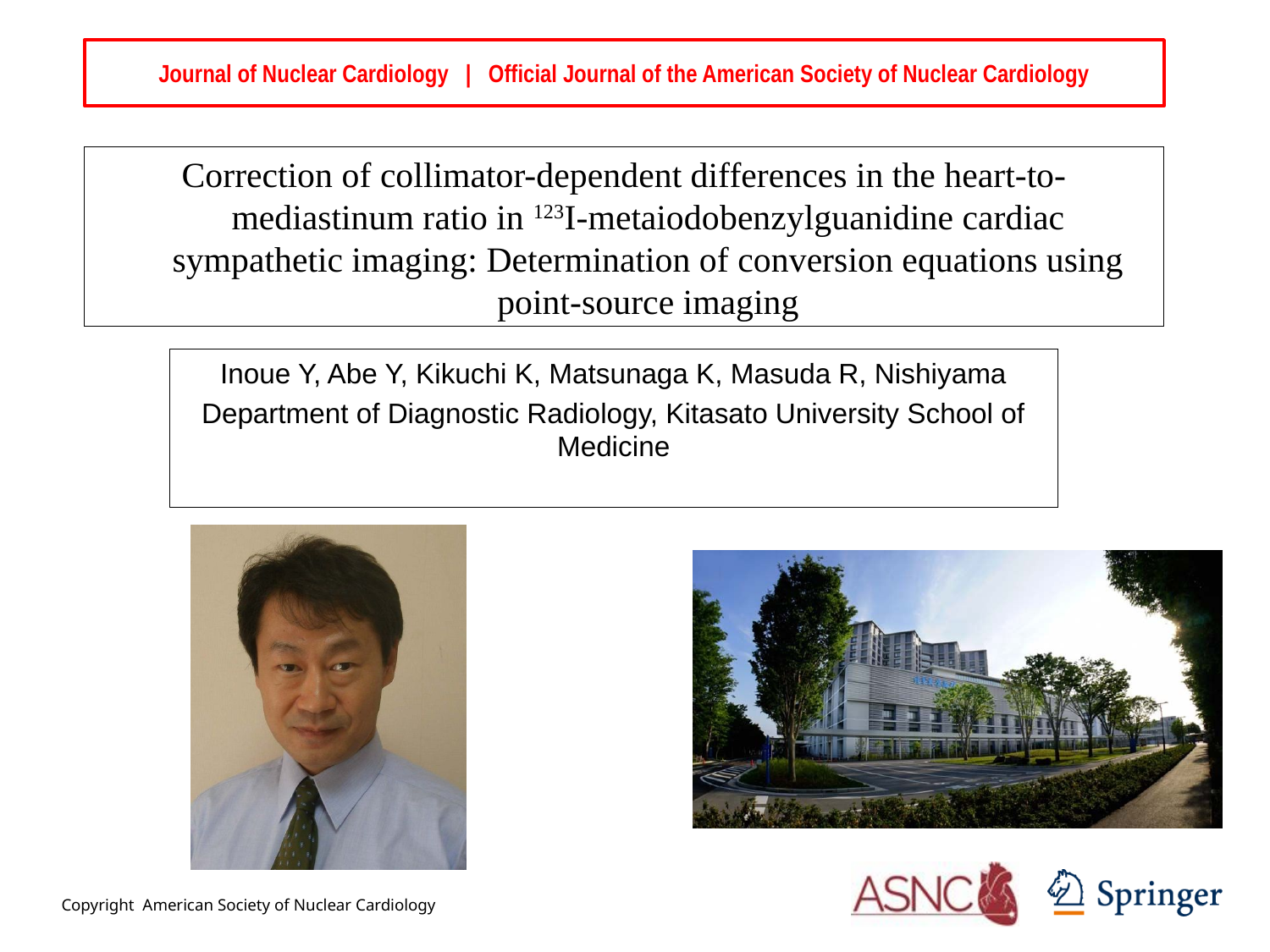

Journal of Nuclear Cardiology | Official Journal of the American Society of Nuclear Cardiology
# Correction of collimator-dependent differences in the heart-to-mediastinum ratio in 123I-metaiodobenzylguanidine cardiac sympathetic imaging: Determination of conversion equations using point-source imaging
Inoue Y, Abe Y, Kikuchi K, Matsunaga K, Masuda R, Nishiyama
Department of Diagnostic Radiology, Kitasato University School of Medicine
Copyright American Society of Nuclear Cardiology

## Slide 2
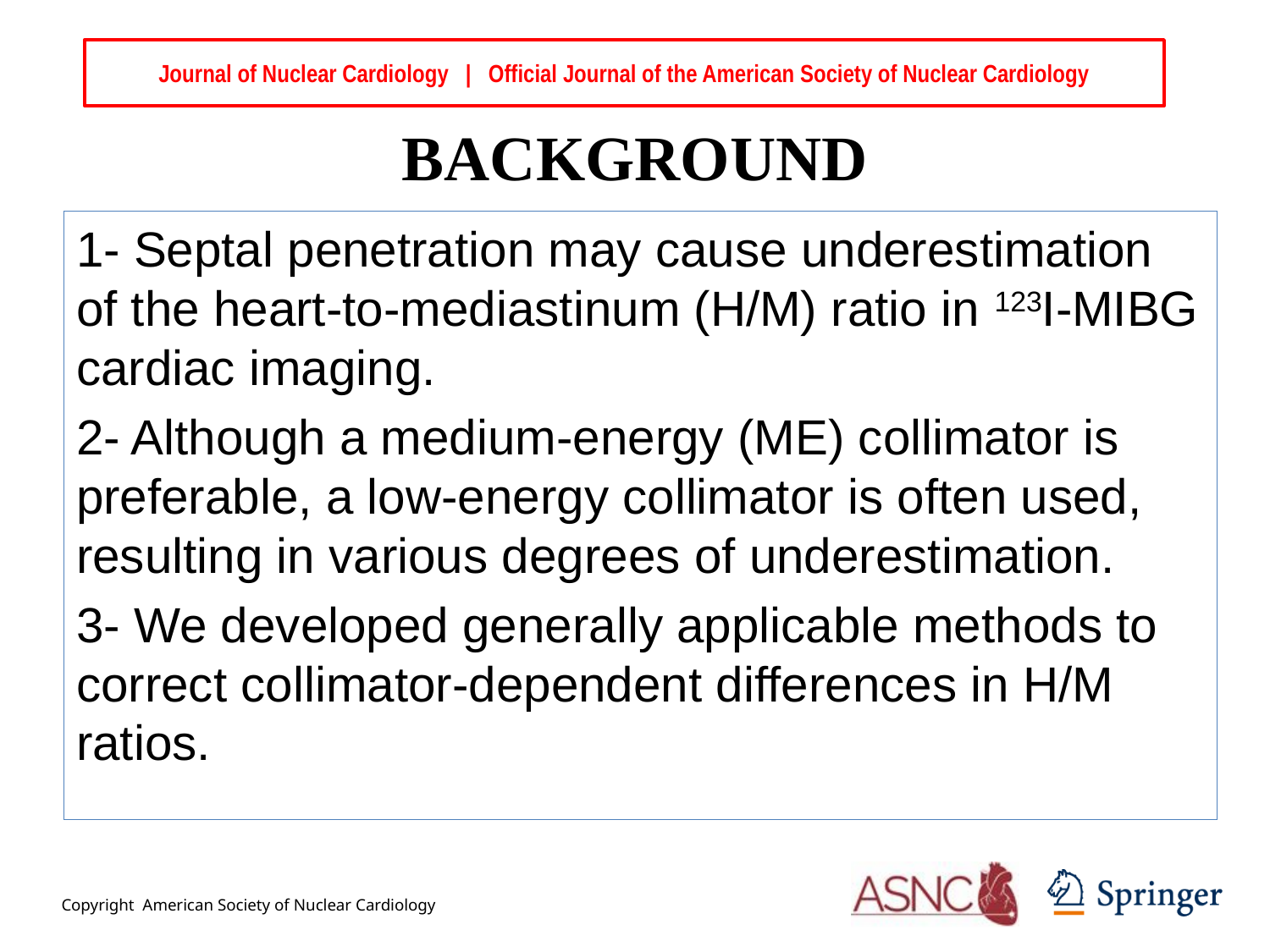

Journal of Nuclear Cardiology | Official Journal of the American Society of Nuclear Cardiology
# BACKGROUND
1- Septal penetration may cause underestimation of the heart-to-mediastinum (H/M) ratio in 123I-MIBG cardiac imaging.
2- Although a medium-energy (ME) collimator is preferable, a low-energy collimator is often used, resulting in various degrees of underestimation.
3- We developed generally applicable methods to correct collimator-dependent differences in H/M ratios.
Copyright American Society of Nuclear Cardiology

## Slide 3
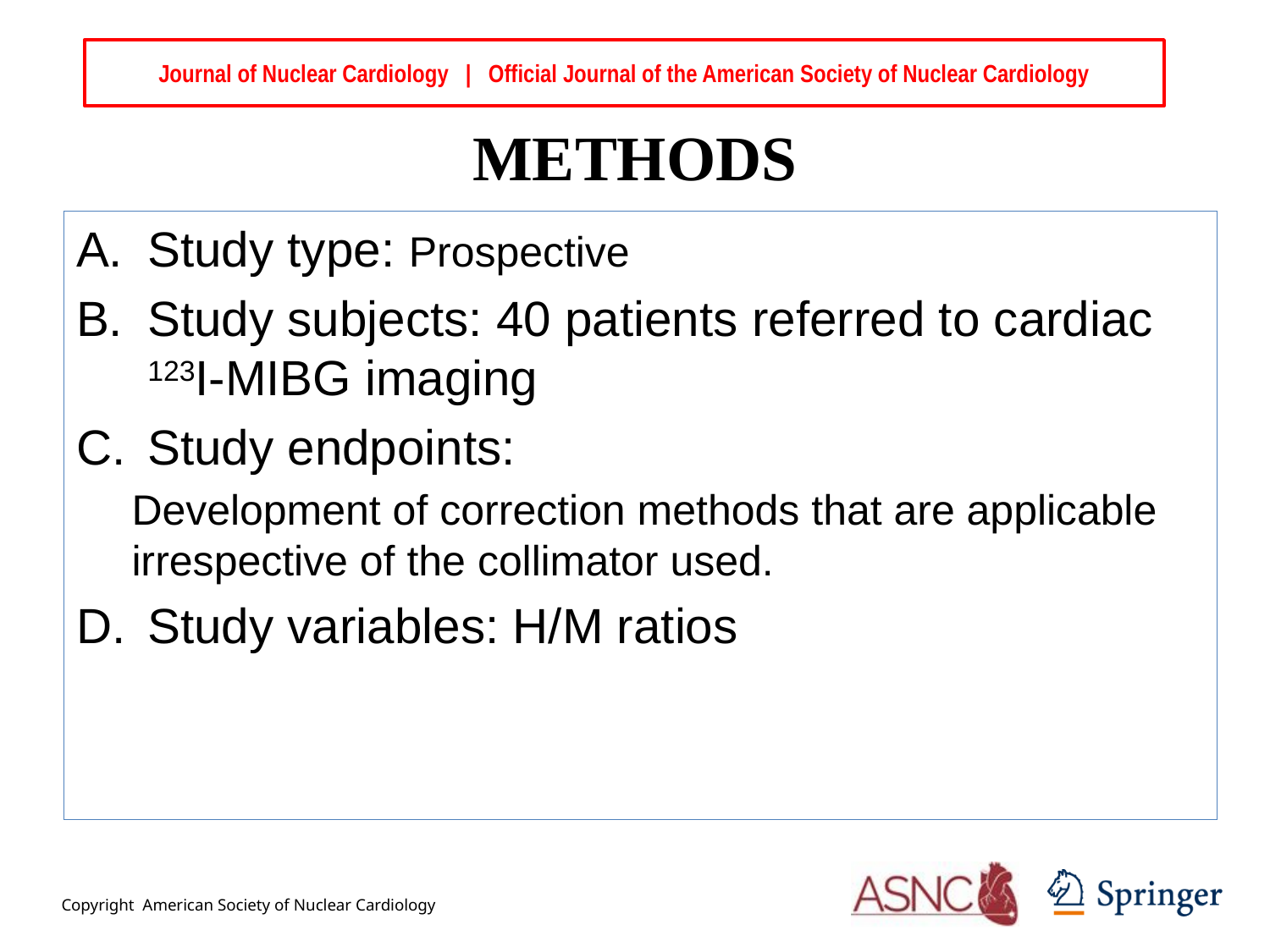

Journal of Nuclear Cardiology | Official Journal of the American Society of Nuclear Cardiology
# METHODS
Study type: Prospective
Study subjects: 40 patients referred to cardiac 123I-MIBG imaging
Study endpoints:
Development of correction methods that are applicable irrespective of the collimator used.
Study variables: H/M ratios
Copyright American Society of Nuclear Cardiology

## Slide 4
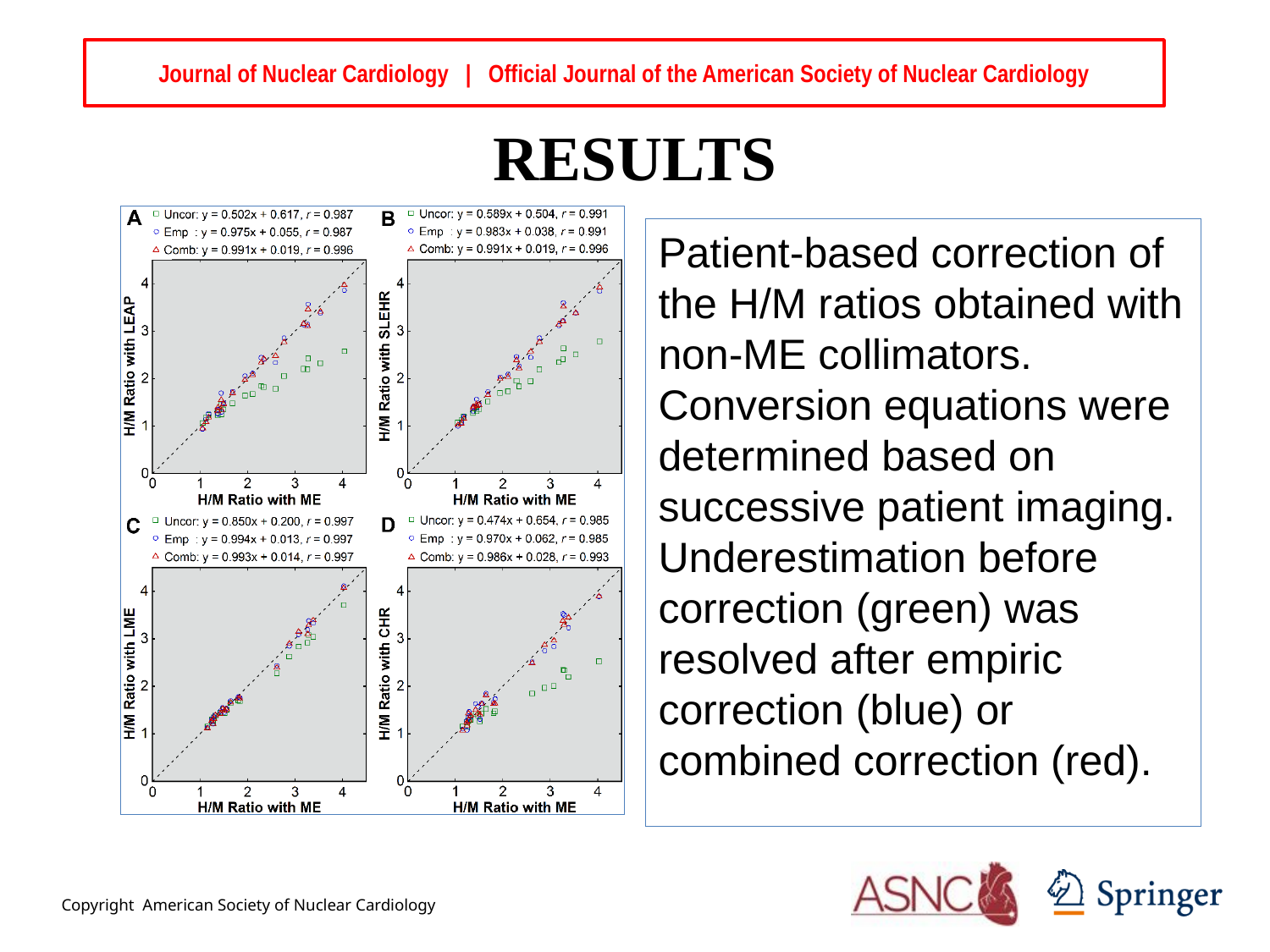

Journal of Nuclear Cardiology | Official Journal of the American Society of Nuclear Cardiology
# RESULTS
Patient-based correction of the H/M ratios obtained with non-ME collimators. Conversion equations were determined based on successive patient imaging. Underestimation before correction (green) was resolved after empiric correction (blue) or combined correction (red).
Copyright American Society of Nuclear Cardiology

## Slide 5
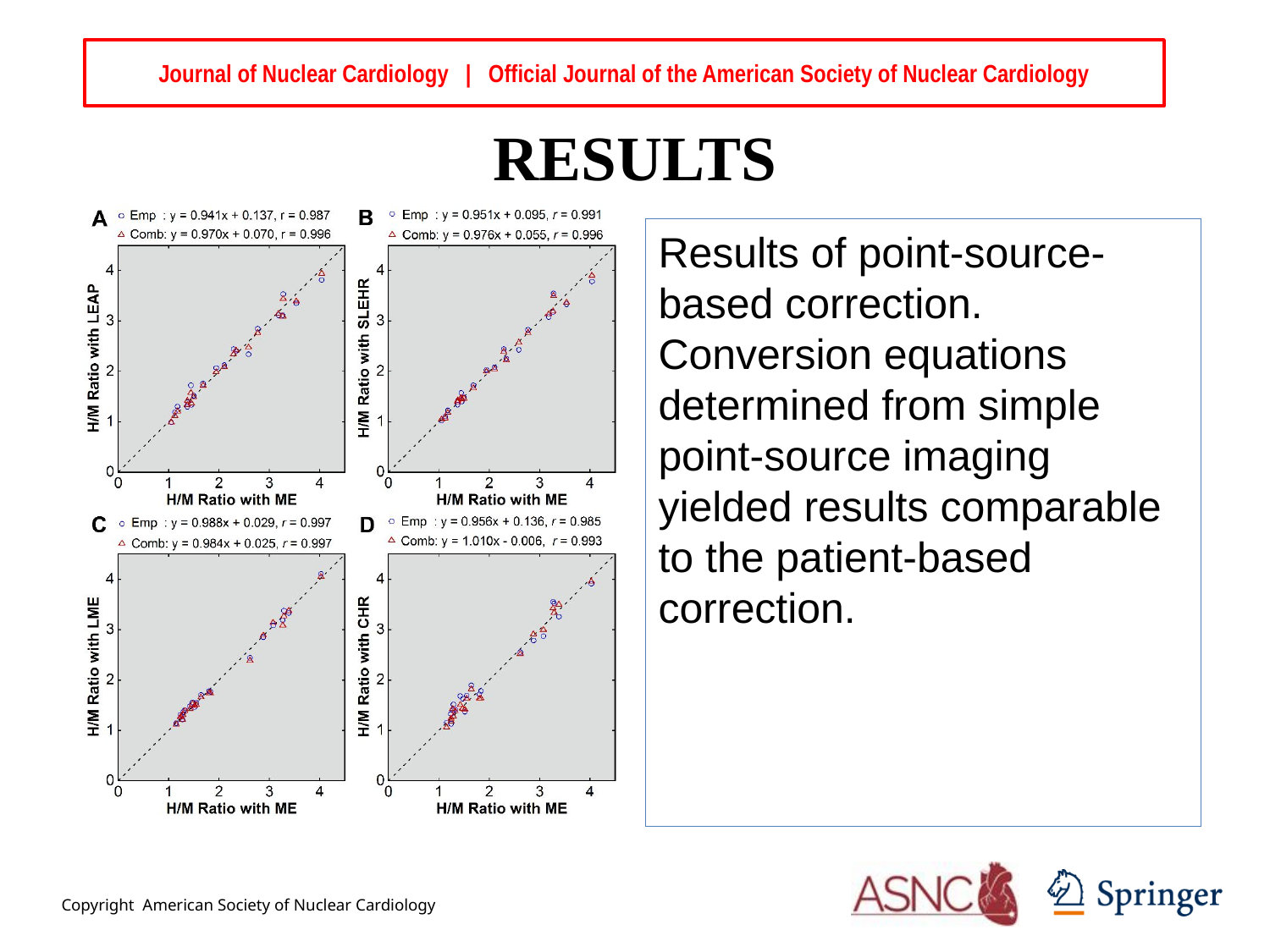

Journal of Nuclear Cardiology | Official Journal of the American Society of Nuclear Cardiology
# RESULTS
Results of point-source-based correction. Conversion equations determined from simple point-source imaging yielded results comparable to the patient-based correction.
Copyright American Society of Nuclear Cardiology

## Slide 6
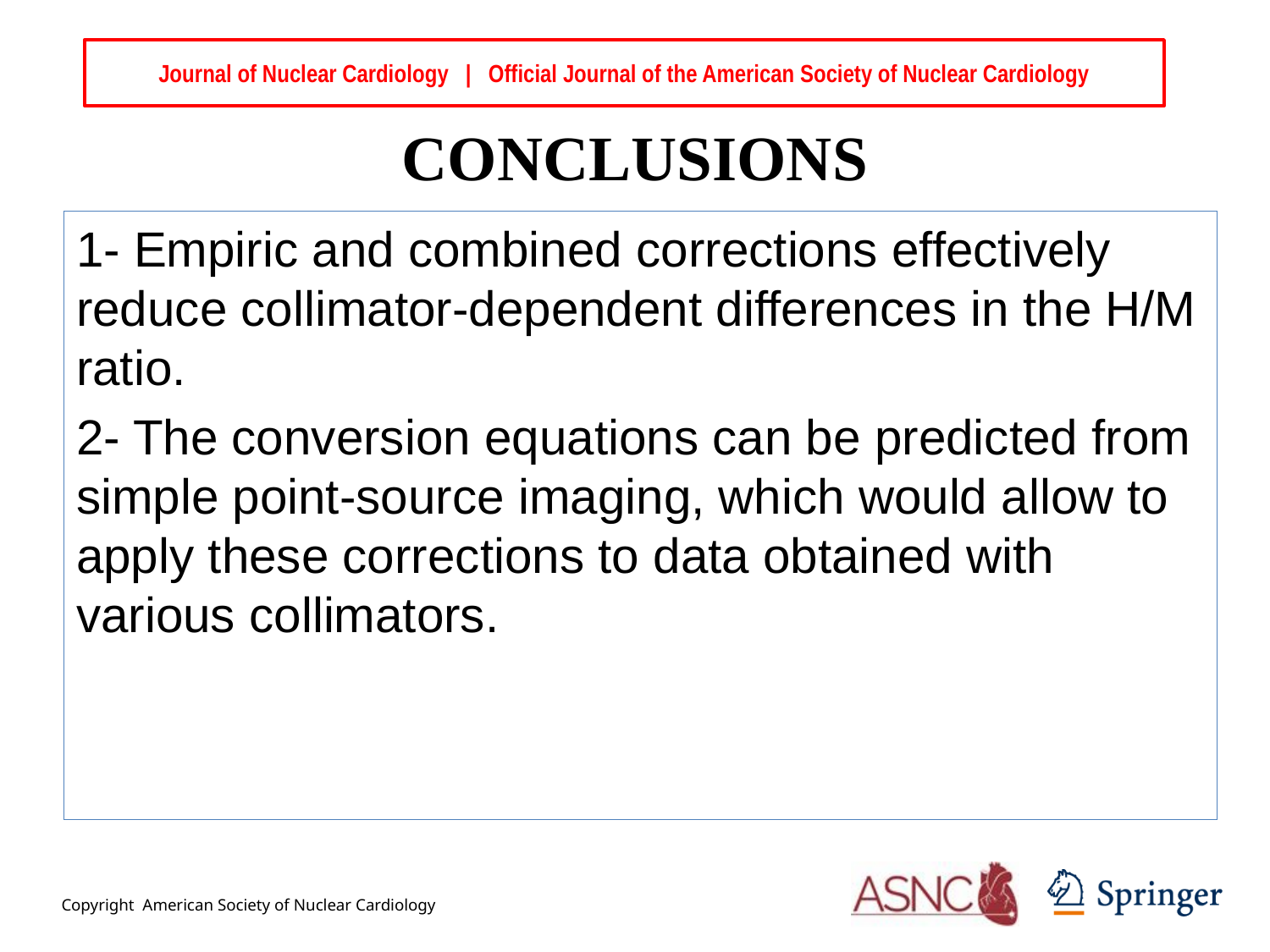

Journal of Nuclear Cardiology | Official Journal of the American Society of Nuclear Cardiology
# CONCLUSIONS
1- Empiric and combined corrections effectively reduce collimator-dependent differences in the H/M ratio.
2- The conversion equations can be predicted from simple point-source imaging, which would allow to apply these corrections to data obtained with various collimators.
Copyright American Society of Nuclear Cardiology

## Slide 7
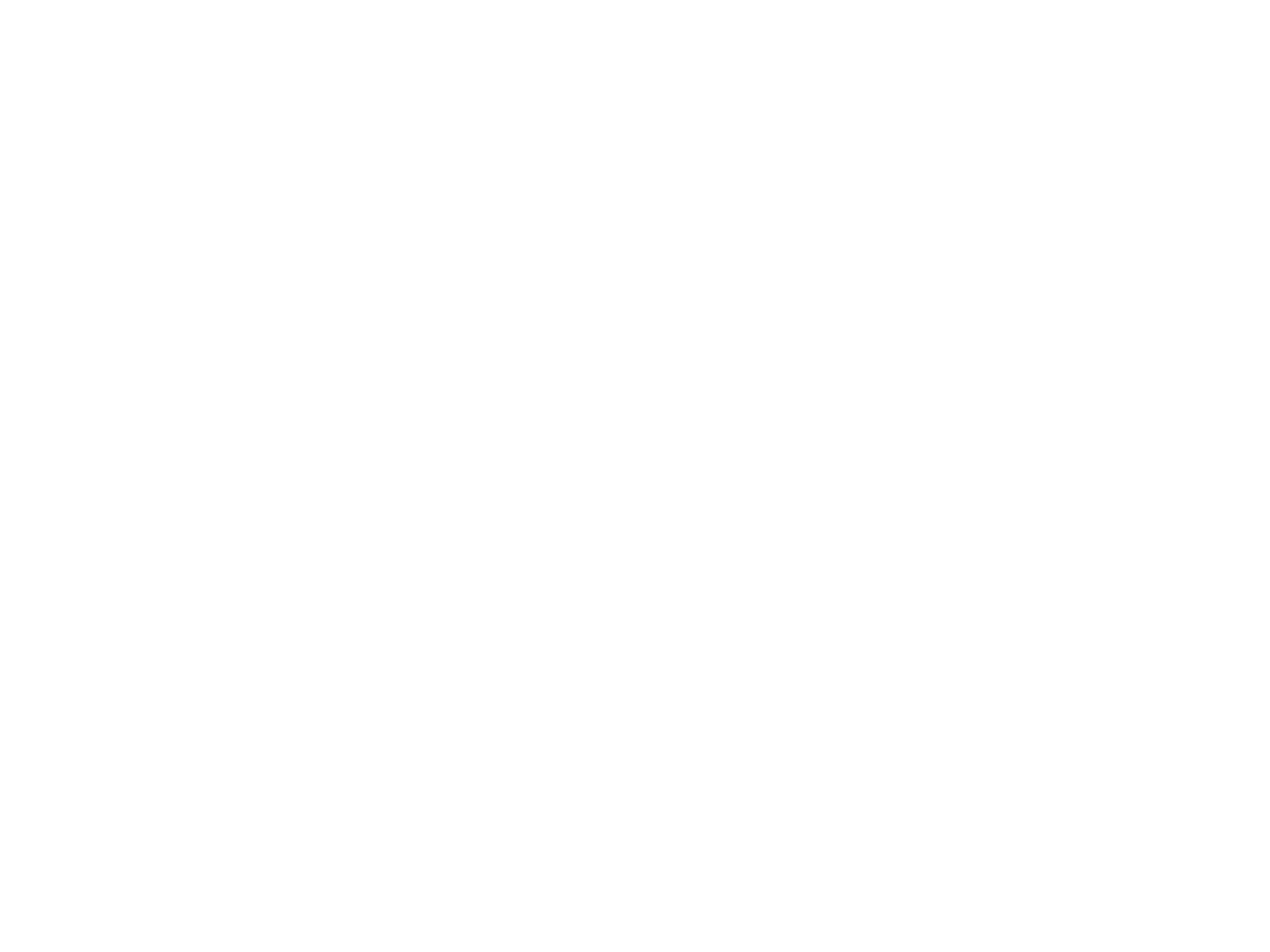

#
